# Non-neutralizing antibodies induced by seasonal influenza vaccine prevent, not exacerbate A(H1N1)pdm09 disease

**DOI:** 10.1038/srep37341

**Published:** 2016-11-16

**Authors:** Jin Hyang Kim, Adrian J. Reber, Amrita Kumar, Patricia Ramos, Gabriel Sica, Nedzad Music, Zhu Guo, Margarita Mishina, James Stevens, Ian A. York, Joshy Jacob, Suryaprakash Sambhara

**Affiliations:** 1 Influenza Division, National Center for Immunization and Respiratory Diseases, Centers for Disease Control and Prevention, 1600 Clifton Rd, Atlanta, GA 30329, USA; 2 Department of Pathology and Laboratory Medicine, School of Medicine, Emory University, 1364 Clifton Rd, N.E. Atlanta, GA 30322, USA; 3 Batelle Memorial Institute, Atlanta, GA 30322, USA; 4 Department of Microbiology and Immunology, Emory Vaccine Center, Yerkes National Primate Center, Emory University, 954 Gatewood Rd, Atlanta, GA, USA

## Abstract

The association of seasonal trivalent influenza vaccine (TIV) with increased infection by 2009 pandemic H1N1 (A(H1N1)pdm09) virus, initially observed in Canada, has elicited numerous investigations on the possibility of vaccine-associated enhanced disease, but the potential mechanisms remain largely unresolved. Here, we investigated if prior immunization with TIV enhanced disease upon A(H1N1)pdm09 infection in mice. We found that A(H1N1)pdm09 infection in TIV-immunized mice did not enhance the disease, as measured by morbidity and mortality. Instead, TIV-immunized mice cleared A(H1N1)pdm09 virus and recovered at an accelerated rate compared to control mice. Prior TIV immunization was associated with potent inflammatory mediators and virus-specific CD8 T cell activation, but efficient immune regulation, partially mediated by IL-10R-signaling, prevented enhanced disease. Furthermore, in contrast to suggested pathological roles, pre-existing non-neutralizing antibodies (NNAbs) were not associated with enhanced virus replication, but rather with promoted antigen presentation through FcR-bearing cells that led to potent activation of virus-specific CD8 T cells. These findings provide new insights into interactions between pre-existing immunity and pandemic viruses.

Emergence of the 2009 swine-origin H1N1 influenza virus, A(H1N1)pdm09, marked the 1^st^ pandemic of the 21^st^ century^1^,^2^. Despite a high disease burden^3^, A(H1N1)pdm09 infection exhibited relatively mild disease comparable to seasonal influenza viruses, resulting in low mortality rates by both US^4^ and world-wide^5^ estimates. However, the epidemiology of A(H1N1)pdm09 infection was different from that of seasonal influenza in its disproportional severity of young adults, with 30–50% of severe or fatal cases concentrated in 30–50 year olds who were otherwise healthy^2^,^3^. Serosurveillance found the presence of pre-existing cross-reactive Abs in older adults >60 years, while pre-pandemic seasonal vaccination induced little cross-reactive Abs against A(H1N1)pdm09, providing explanations to its infection pattern^6^,^7^. While most epidemiology studies supported the serological findings that the seasonal trivalent inactivated vaccine (TIV) conferred either partial or no protection against A(H1N1)pdm09 infection^8^,^9^,^10^,^11^, other studies including a Canadian sentinel study found an unexpected association of seasonal TIV with increased risk of A(H1N1)pdm09 illness^12^,^13^,^14^ even after adjustment for several confounding factors^13^,^15^. Several biological mechanisms including original antigenic sin, antibody-dependent enhancement of infection (ADE) or temporary heterosubtypic immunity conferred by seasonal influenza infection have been proposed for the increased risk^16^,^17^,^18^, but its cause remains unknown.

The findings from the Canadian sentinel studies have inspired animal studies for validation and investigation of mechanisms for the observed finding in humanss^19^,^20^,^21^,^22^. Some studies demonstrated vaccine-associated enhanced disease, a phenomenon that has been described for respiratory syncytial virus (RSV)^23^ and measles virus^24^ infections following vaccination with whole-inactivated viruses (WIV). Molecular mechanisms of vaccine-associated enhanced disease remain largely unknown; although the involvement of immune complex (IC)-mediated complement activation and B cells have been proposed for RSV infection^25^. In mismatched influenza infection of pigs, wherein prior vaccination with human-like H1N2 WIV worsened A(H1N1)pdm09 disease, hemagglutinin (HA)-binding, non-neutralizing Abs (NNAbs) were sufficient to cause vaccine-associated enhanced respiratory disease (VAERD)^26^ by promoting virus membrane fusion, independently of Fc receptor (FcR)-mediated endocytosis^22^. These findings provide a potential mechanism for the association of seasonal TIV and increased risk of A(H1N1)pdm09 illness. However, caution needs to be taken in interpreting and applying these animal findings to human A(H1N1)pdm09 infections. Since the findings of Canadian sentinel study were reported, four additional observational studies confirmed TIV as a risk factor for medically attended A(H1N1)pdm09 illness; however, these studies consistently found that TIV did not increase the severity of disease^13^,^16^. On the contrary, studies examining hospitalized cases including intensive care unit admission or death, found the strongest association with pre-existing medical conditions, not seasonal TIV immunization^27^. Therefore, considering the nature of epidemiological and observational studies, it is possible that A(H1N1)pdm09 infection caused enough malaise or discomfort in TIV-recipients to seek medical attention, yet did not lead to severe outcomes. Vaccine-associated enhanced disease remains a possible reason for increased medical attention seeking of TIV-recipients upon A(H1N1)pdm09 infection. This was indeed tested in ferrets recently; however, the majority (94%) of TIV-immunized ferrets failed to develop detectable hemagglutination inhibition (HI) titers prior to A(H1N1)pdm09 infection^28^, making it difficult to compare to the clinical findings of human A(H1N1)pdm09 infections and to accurately assess the direct vaccine effect.

Here, we sought to investigate if A(H1N1)pdm 09 infection enhanced disease in TIV-primed mice and if so, identify the possible underlying mechanisms. To this end, mice were first immunized with seasonal TIV (A/Brisbane/59/2006 (Bris59; H1N1), A/Brisbane/10/2007 (H3N2), B/Brisbane/60/2008), then infected with A/California/08/09 (Cal08, a A(H1N1)pdm09 virus) and monitored for morbidity and mortality as primary disease outcomes. TIV-induced Abs were predominantly NNAbs, based on *in vitro* serological assays, yet infection of TIV-immune mice with Cal08 virus was attenuated. Despite reduced virus titers, Cal08 infection in TIV-immune mice elicited potent inflammatory mediators and virus-specific CD8 T cell responses. However, instead of exacerbation of morbidity and/or mortality, TIV/Cal08 mice showed accelerated viral clearance and recovery compared to PBS/Cal08 mice. Prevention of aberrant morbidity was partially mediated by IL-10R signaling. Potent CD8 T cell activation was associated with CD11c^hi^ cells containing intracellular nucleoprotein (NP), which was facilitated by NNAb, suggesting a role of NNAbs-mediated cross-presentation for protection. Therefore, our findings indicate that prior seasonal immunization did not enhance A(H1N1)pdm09 virus disease, but elicited protective immunity through mechanisms similar to those described for heterosubtypic immunity.

## Results

### TIV-immunization induced non-neutralizing yet cross-reactive, weak-binding Abs that attenuated Cal08 infection

**Figure 1:**
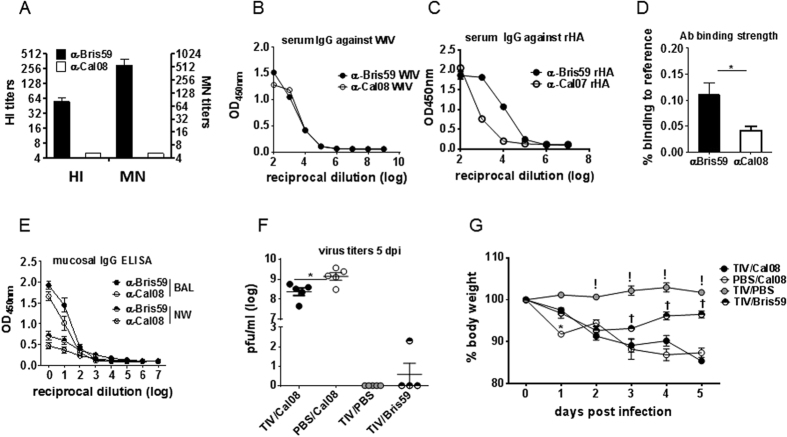
TIV-immunization induced non-neutralizing yet cross-reactive, weak-binding Abs that attenuated Cal08 infection. (**A**–**E**) Balb/c mice (5 mice/group) were i.m. immunized with 9 μg TIV/100 μl or PBS. At d30 post-immunization, the sera, BAL and nasal washes were collected for characterization of TIV-induced Abs. (**A**) Functional neutralizing activity of sera was measured by HI and MN titers against wild-type Bris59 or Cal08 virus. (**B**,**C**) Virus-binding ability of sera was measured by ELISA against WIV Bris59 or Cal08 virus or His-tagged rHA Bris59 or Cal07 proteins. (**D**) Sera Ab binding strength for Bris59 or Cal08 virus were measured by biolayer interferometry. Student t-test was used for comparison of titers between the 2 groups (*P* < 0.05) (**E**) Mucosal surface (BAL and nasal washes) Ab responses were measured by ELISA against WIV Bris59 or Cal08 virus. (**F**,**G**) TIV or mock (PBS)-immunized mice (5 mice/group) were infected with 5 × 10^6^pfu Cal08 virus (TIV/Cal08, PBS/Cal08 group) or Bris59 virus (TIV/Bris59 group) at d > 30 post-immunization. Control mice were TIV-immunized, mock-infected (TIV/PBS group). All mice were sacrificed at d5 post-Cal08 infection. (**F**) Lung virus titers at 5 dpi were assessed via plaque assay on MDCK cells. Unpaired t test with Welch’s correction was used to compare TIV/Cal08 vs. PBS/Cal08 groups (*P* < 0.05), as data points in TIV/PBS and TIV/Bris59 could not be transformed for one-way ANOVA. (**G**) BW was monitored for 5 days post-infection. Two-way ANOVA with Bonferroni’s multiple comparison test was used for *P* values. *Comparison between TIV/Cal08 vs. PBS/Cal08 groups (*P* < 0.001), ! comparison between TIV/Cal08 vs. TIV/PBS groups (*P* < 0.001), ^†^Comparison between TIV/Cal08 vs. TIV/Bris59 groups (*P* < 0.001). The error bars represent standard error of the mean (SEM). The data are a representative of 5 experiments.

To understand the impact of seasonal TIV on A(H1N1)pdm09 infection, TIV-immune sera collected at d30 post-immunization were first evaluated for function and binding characteristics (Fig. 1A–E). TIV-sera were non-neutralizing against Cal08 (undetectable HI and MN titers) (Fig. 1A), yet capable of binding to Cal08 WIV or rHA at comparable or modestly reduced levels to Bris59 WIV or rHA, respectively (ELISA titers) (Fig. 1B,C). However, the binding strength for Cal08 was significantly weaker compared to Bris59, shown by biolayer interferometry (Fig. 1D). Mucosal IgG responses (BAL and nasal washes) measured by ELISA, were also comparable against both viruses (Fig. 1E). Because of NNAbs, virus replication in TIV-mice was expected to be comparable to that in PBS/Cal08 mice. However, upon Cal08 (5 × 10^6^pfu) infection, TIV-mice showed modestly, yet significantly lower lung virus titers compared to PBS/Cal08 controls (2.4 × 10^8^ vs. 1.4 × 10^9^ pfu/ml, *P* = 0.02) at 5 days post-infection (dpi) (Fig. 1F). Despite reduced virus titers, the morbidity as measured by body weight (BW) loss in TIV/Cal08 mice was similar to that of PBS/Cal08 mice (≤15% original BW). However, TIV-mice infected with Bris59 virus (vaccine-match control) efficiently cleared virus, and started to recover by 2dpi (Fig. 1G).

### Pro-inflammatory mediators were significantly elevated in TIV/Cal08 mice

**Figure 2:**
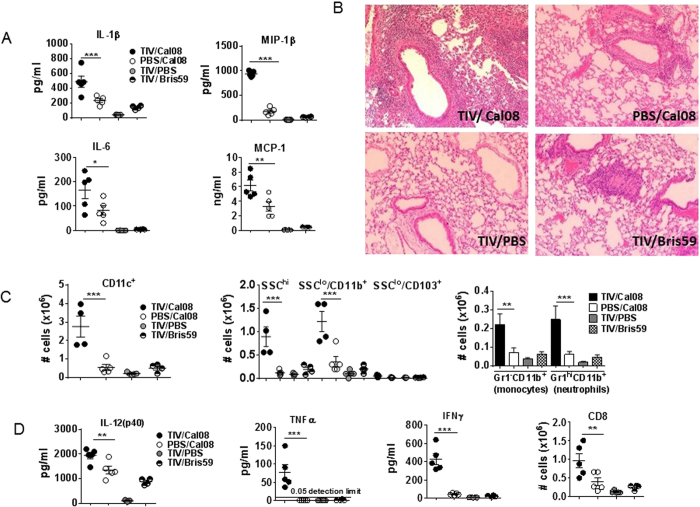
Pro-inflammatory mediators were significantly elevated in TIV/Cal08 mice. TIV or mock-immunized mice (4–5 mice/group) were infected with 5 × 10^6^pfu Cal08, or Bris59 virus or mock-infected. Lung lysates were collected at d5 post-infection. (**A**) Lung lysates of TIV/Cal08, PBS/Cal08, TIV/PBS or TIV/Bris59 mice were analyzed for the level of pro-inflammatory mediators (IL-1β, Mip-1β, IL-6, MCP-1) by Bio-Plex^TM^. (**B**) Whole lung tissues from a representative mouse of each group were fixed with 10% formalin for histology (H&E staining). (**C**) Lung single cell suspensions were analyzed for the frequency of CD11c^+^ and their subsets (SSC^hi^, SSC^lo^/CD11b^+^, SSC^lo^/CD103^+^), and CD11b^+^ and their subsets (inflammatory monocytes: Gr1^+^ CD11b^+^ SSC^int^ and neutrophils:CD11c^−^CD11b^+^ Gr1^hi^) by flow cytometry and their numbers were calculated from lung cell counts. (**D**) IL-12, TNFα, IFNγ in lung lysates were analyzed by Bio-Plex^TM^ and the frequency of CD8 T cells was measured by flow cytometry. The error bars represent standard error of the mean (SEM). One-way ANOVA with Bonferroni post-test was used to calculate the *P* values (*P < 0.05; **P < 0.01; ***P < 0.001). The data are a representative of 3 experiments.

Since virus titers of TIV/Cal08 mice were reduced, lung inflammatory mediators were expected to be reduced. Surprisingly however, pro-inflammatory mediators (IL-1β, MIP-1β, IL-6, MCP-1) were significantly elevated in TIV/Cal08 compared to PBS/Cal08 mice at 5dpi, while low in TIV/PBS and TIV/Bris59 mice (Fig. 2A). Lung histology of TIV/Cal08 mice also revealed significant mononuclear cell infiltration compared to controls (Fig. 2B) and scored highest in lymphocyte infiltration as well as small airway and bronchiolar inflammation (Supplemental Fig. 1A). Flow cytometric analysis revealed significant infiltration of phagocytic cells including subsets of CD11c^+^ cells and CD11b^+^ cells in TIV/Cal08 mice’s lungs compared to controls both in absolute cell numbers and percent (Fig. 2C, Supplemental Fig. 1B,C). Cytokines associated with T cell effector function (IL-12p40, TNFα, IFNγ) (Fig. 2D) and chemokine associated with lymphocyte recruitment (IP-10) (Supplemental Fig. 1D) were also significantly elevated in TIV/Cal08 mice compared to controls. Accordingly, significantly more CD8 T cells (Fig. 2D), B cells and their Ig class-switched B cell subsets, follicular helper T cells (T_FH_) (Supplemental Fig. 2D) were found in lungs of TIV/Cal08 mice compared to controls.

### Virus-specific CD8 T cells were significantly induced in TIV/Cal08 mice

**Figure 3:**
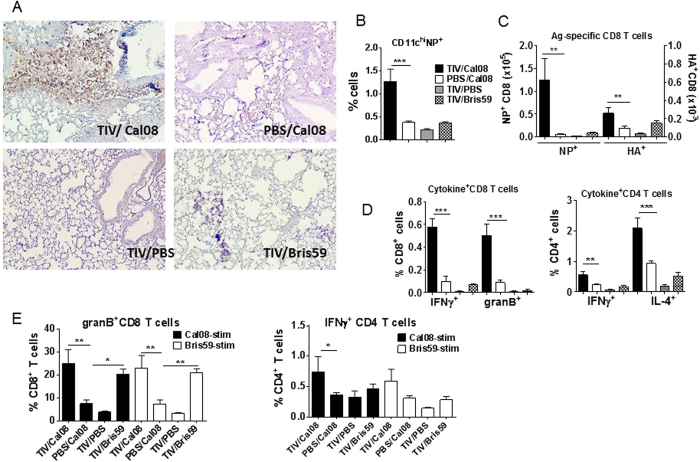
Virus-specific CD8 T cells were significantly induced in TIV/Cal08 mice. TIV or mock-immunized mice (4–5 mice/group) were infected with 5 × 10^6^pfu Cal08, or Bris59 virus or mock-infected. All mice were sacrificed at d5 post-infection. (**A**) Sections of formalin-fixed lung tissues at 5 dpi were stained via immunochemistry for NP signals. (**B**) Lung single cell suspensions at 5 dpi were intracellularly stained for NP via flow cytometry and the %CD11c^hi^ cells that stained for NP are shown. (**C**) Virus-specific CD8 T cells were stained with NP- or HA-pentamers for flow cytometry and their cell numbers were calculated from total lung cell counts. (**D**) CD8 or CD4 T cells secreting IFNγ, granB, or IL-4 *ex vivo* were analyzed by intracellular staining of cytokines without *in vitro* stimulation. (**E**) Virus-specific CD8 or CD4 T cell responses were assessed by *in vitro* stimulation with Cal08 or Bris59 virus at a multiplicity of infection (MOI) of 1 overnight and subsequent surface and intracellular cytokine staining for flow cytometry. The error bars represent standard error of the mean (SEM). One-way ANOVA with Bonferroni post-test was used to calculate the *P* values (*P < 0.05; **P < 0.01; ***P < 0.001). The data are a representative of 3 experiments.

Reduction of virus titers upon infection primarily depends on pre-existing, neutralizing Abs. However, NNAbs raised against nuclear protein (NP) have also been shown to protect mice from lethal infection^29^,^30^,^31^,^32^,^33^. In particular, LaMere and colleagues have shown that protection through NP-reactive NNAbs involves CD8 T cells and FcR^34^. Furthermore, León and colleagues have demonstrated that NP-immune complexes prolong cross-presentation by DCs to CD8 T cells^35^. Therefore, reduced virus titers in TIV/Cal08 mice compared to PBS/Cal08 mice, despite the non-neutralizing property of TIV-induced Abs (Fig. 1), indicate a possible involvement of immune complexes, FcR and memory CD8 T cells during the secondary responses. Indeed, FcγR (CD32/16) of TIV/Cal08 mice was significantly upregulated in all CD11b^+^ cells, except neutrophils (Supplemental Fig. 2A). Further, immunohistochemistry of lung sections revealed visually higher NP staining in TIV/Cal08 mice compared to controls (Fig. 3A), suggesting that NNAbs were associated with higher intracellular NP levels. Accordingly, flow cytometric analysis showed that subsets of phagocytic cells (CD11c^hi^ and CD11b^+^ Ly6C^lo^ cells) of TIV/Cal08 mice contained significantly more intracellular NP signals than PBS/Cal08 mice (Fig. 3B, Supplemental Fig. 2B,C). Higher viral protein signals in APCs were associated with significant recruitment of NP- or HA-pentamer-specific CD8 T cells (Fig. 3C). In addition, secretion of IFNγ, granzyme-B (granB) or IL-4 by T cells was readily detected *ex vivo* as well as upon *in vitro* stimulation in TIV/Cal08 mice (Fig. 3D,E, Supplemental Fig. 2D,E). Comparable granB, TNFα or IFNγ-secretion upon stimulation indicates significant conservation of T cell epitopes between Cal08 and Bris59 viruses. These data suggest a significant role for FcR-bearing cells in virus-uptake and subsequent T cell activation in TIV/Cal08 mice.

### IL-10R signaling prevented disease exacerbation in TIV/Cal08 mice

**Figure 4:**
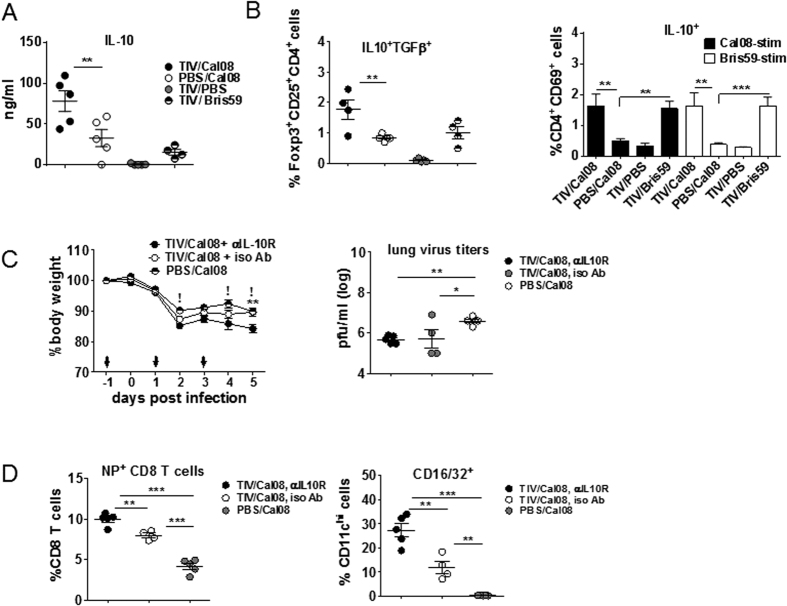
IL-10R signaling prevented disease exacerbation in TIV/Cal08 mice. TIV or mock-immunized mice (4–5 mice/group) were infected with 5 × 10^6^pfu Cal08 or Bris59 virus or mock-infected. All mice were sacrificed at d5 post-infection. (**A**) IL-10 in lung lysates was measured by Bio-Plex^TM^. (**B**) Lung single cell suspensions were *ex vivo* stained for CD4^+^ CD25^+^ Foxp3^+^ cells secreting both IL-10 and TGFβ. Cells were also *in vitro-*stimulated with Cal08 or Bris59 virus at MOI 1 overnight for measuring IL-10-secreting CD4 T cells by flow cytometry. (**C**,**D**) TIV-mice (4–5 mice/group) were injected with 0.5 mg/mouse IL-10R blocking Ab (αIL-10R) or isotype control Abs on days −1, +1 and +3 post-infection (arrows). Mock-immunized mice were control. All mice were infected with 5 × 10^6^pfu/mouse Cal08 virus and sacrificed at d5 post-infection. (**C**) BWs were monitored for 5 days post-infection and lung virus titers at 5 dpi were measured via plaque assay. Two-way ANOVA with Bonferroni’s multiple comparison test was used for *P* values. **Comparison between TIV/Cal08 + αIL-10R Ab vs. TIV/Cal08 + iso Ab groups (*P* < 0.01), ! comparison between TIV/Cal08 αIL-10R Ab vs. PBS/Cal08 groups (*P* < 0.01). (**D**) Lung cells were stained with NP pentamers for assessment of virus-specific CD8 T cells or a high level of CD16/32 expressing CD11c^hi^ cells via flow cytometry. The error bars represent standard error of the mean (SEM). All comparisons except (**C**) were done by One-way ANOVA with Bonferroni post-test. *P < 0.05; **P < 0.01; ***P < 0.001. The data (**A**,**B**) are a representative of 3 experiments and data (**C,D**) are a representative of 2 experiments.

Upregulation of pro-inflammatory mediators and potent CD8 T cell activation could potentially lead to immunopathology, yet comparable morbidity between TIV/Cal08 and PBS/Cal08 mice (Fig. 1G) indicates a counteracting mechanism at play. Previous studies have shown that lung inflammation is controlled by IL-10 produced by CD4 and CD8 T cells during acute primary influenza infection^36^. Therefore, IL-10 may contribute to balanced immune responses in TIV/Cal08 mice. Accordingly, lung IL-10 was significantly elevated in TIV/Cal08 compared to control groups (Fig. 4A). Further, significantly higher IL-10, TGFβ-secretion from Treg (CD4^+^ CD25^+^ Foxp3^+^) cells *ex vivo* as well as IL-10 secretion from *in vitro-*stimulated CD4 T cells were detected in TIV/Cal08 mice compared to controls (Fig. 4B). To further investigate the role of IL-10R signaling in TIV/Cal08 mice, TIV-mice were treated with 0.5 mg IL-10R-blocking or isotype control Abs on −1, +1 and 3 dpi. Upon Cal08 infection, αIL-10R-TIV/Cal08 mice lost more BW than control Ab-TIV/Cal08 mice or PBS/Cal08 mice at all time-points and was significant at 5 dpi (Fig. 4C). However, this was not due to a direct suppression of virus replication, as virus titers were comparable regardless of IL-10R-blocking in TIV/Cal08 mice (Fig. 4C). Despite similar virus titers, αIL-10R-treated mice showed significantly higher NP-specific CD8 T cells and FcγR expression in CD11c^hi^ cells compared to control Ab-treated mice (Fig. 4D), suggesting that regulation by IL-10R signaling may operate at the level of cross-presentation. Together, these data suggest that regulatory mechanism partially mediated by IL-10R signaling prevented severe outcomes in TIV/Cal08 mice.

### TIV-immunized mice cleared Cal08 virus and recovered faster than controls following infection

**Figure 5:**
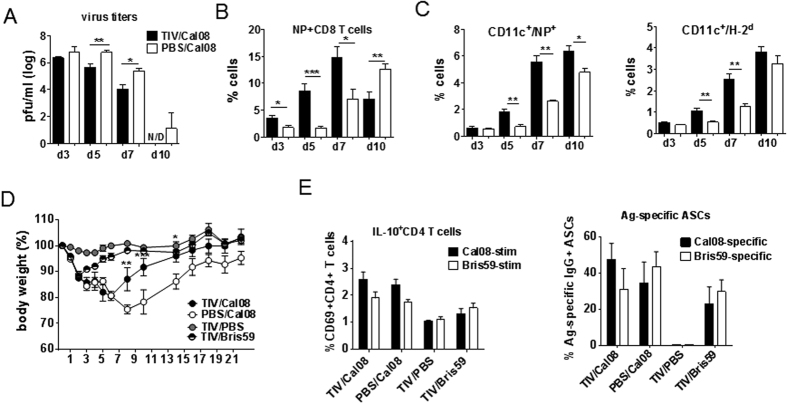
TIV-immunized mice cleared Cal08 virus and recovered faster than controls following infection. (**A–C**) TIV or mock-immunized mice (4–5 mice/group were infected with 5 × 10^6^pfu Cal08 virus. Four or five mice were sacrificed at d3, 5, 7 and 10 post-infection. (**A**) Lung virus titers at days 3, 5, 7 and 10 post-infection were assessed via plaque assay. (**B**) Lung cells were stained with NP pentamers to assess virus-specific CD8 T cells via flow cytometry at days 3, 5, 7 and 10 post-infection. (**C**) CD11c^hi^ cells with intracellular NP signal or expressing high levels of MHC class I (H-2^d^) were measured via flow cytometry at days 3, 5, 7 and 10 post-infection. (**D**,**E**) TIV or mock-immunized mice were infected with 5 × 10^6^pfu Cal08 or Bris59 virus or mock-infected. (**D**) BW was monitored until 21 dpi. Two-way ANOVA with Bonferroni’s multiple comparison test was used to compare multiple groups. Comparison between TIV/Cal08 vs. PBS/Cal08 mice is shown. (**E**) At 21 dpi, mice were sacrificed and splenocytes were stimulated *in vitro* with Cal08 or Bris59 virus at MOI 1 for measuring IL-10-secreting CD4 T cells, or placed onto ELISPOT plates to measure ASCs specific to Cal08 or Bris59 virus. N/D; not detectable. The error bars represent standard error of the mean (SEM). All comparisons except (**D**) were done by One-way ANOVA with Bonferroni post-test. *P < 0.05; **P < 0.01; ***P < 0.001. The data are a representative of 2 experiments.

Heightened, yet tightly-regulated immune responses of TIV/Cal08 mice not only protected the mice from disease exacerbation, but also promoted viral clearance, which correlated with accelerated kinetics of virus-specific CD8 T cell responses (Fig. 5A,B, Supplemental Fig. 3A). While NP-specific CD8 T cells of PBS/Cal08 mice gradually reached peak levels at 10dpi, TIV/Cal08 mice demonstrated significantly higher NP-specific CD8 T cells at 3 dpi, peaked at 7 dpi, then contracted at 10 dpi. Accelerated CD8 T cell activation, in turn, correlated with accelerated induction of CD11c^hi^ cells and their accumulation of intracellular NP signals as well as upregulation of MHC class I (H-2^d^) and MHC class II (I-A^d^) (Fig. 5C, Supplemental Fig. 3B). Consequently, TIV/Cal08 mice also recovered faster than PBS/Cal08 mice (Fig. 5D). While TIV/Cal08 mice fully reached the BW level of TIV or TIV/Bris59 mice by 13 dpi, PBS/Cal08 mice’s BW remained lower at all time-points and failed to reach the original BW even at 21 dpi. The immune-homeostasis was also restored by 21 dpi, as IL-10^+^ CD4 T cells (Fig. 5E) or granB^+^ CD8 T cells (Supplemental Fig. 3C) that were acutely elevated at 5 dpi, became comparable between TIV/Cal08 and PBS/Cal08 mice at 21 dpi. Interestingly, virus-specific antibody-secreting cells (ASCs) were developed in all infected mice at a comparable level between TIV/Cal08 and PBS/Cal08 mice (Fig. 5E), indicating no evidence of original antigenic sin. Consistently, B cells undergoing germinal center (GC) reaction were comparable between the two groups (Supplemental Fig. 3C).

### NNAbs were not associated with enhanced disease

**Figure 6:**
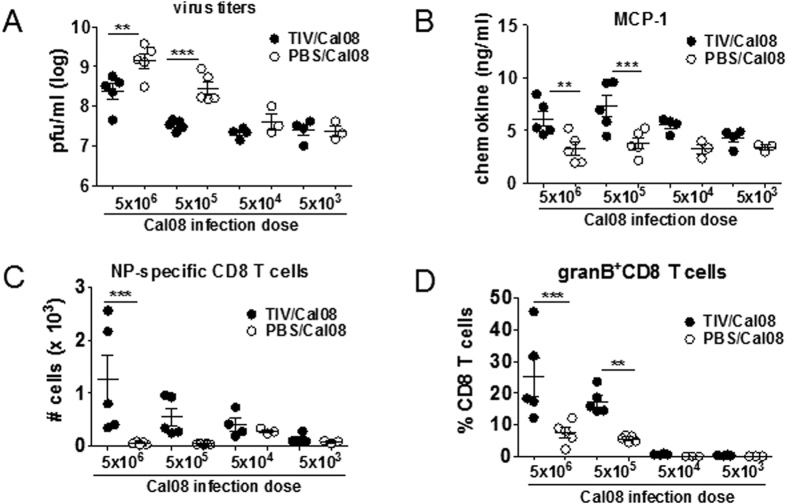
NNAbs were not associated with enhanced disease. TIV- or mock-immunized Balb/c mice (4–5 mice/group) were infected with 5 × 10^3^-5 × 10^6^pfu/mouse Cal08 virus in 1 log increments at d > 30 post-immunization. (**A**) Lung virus titers at 5 dpi were analyzed via plaque assay. (**B**) Lung lysates were analyzed for the level of the pro-inflammatory mediator, MCP-1 via Bio-Plex^TM^. (**C**) Lung cells were stained with NP pentamers to measure virus-specific CD8 T cells via flow cytometry. (**D**) Lung CD8 T cells secreting granB *ex vivo* were measured via flow cytometry. The error bars represent standard error of the mean (SEM). One-way ANOVA with Bonferroni post-test was used to calculate the *P* values (*P < 0.05; **P < 0.01; ***P < 0.001). The data are a representative of 2 experiments.

Recent studies on VAERD in the pig model showed a pathological role of HA2-binding NNAbs in promoting viral membrane fusion of A(H1N1)pdm09 virus^22^. In addition, severe human A(H1N1)pdm09 cases were correlated with high levels of NNAbs with low avidity and complement-activating activity^37^,^38^. In the current study, however, the mere presence of NNAbs was not sufficient to lead to severe A(H1N1)pdm09 disease and the complement depletion did not change the disease course (Discussion). To test if TIV-associated A(H1N1)pdm09 disease could be manifested by differential Ag/Ab ratios, TIV-mice were infected with sub-lethal doses (5 × 10^3^–5 × 10^6^pfu) of Cal08 virus as an attempt to increase the NNAbs/virus ratio. All TIV-immune mice lost BW proportionally to infection dose at a range of 4–16% of the original BW, yet disease was not precipitated at any dose (Supplemental Fig. 4A). Further, lung virus titers at 5dpi were either lower (at 5 × 10^5^–5 × 10^6^pfu/mouse) than or equal (at 5 × 10^3^–5 × 10^4^pfu/mouse) to those of PBS/Cal08 mice (Fig. 6A). Despite reduced virus titers, several inflammatory mediators including MCP-1, IL-1β, IL-6 and NP-specific CD8 T cells, granB^+^ CD8 T cells and CD11c^hi^ cells were elevated in TIV/Cal08 compared to PBS/Cal08 mice at the two higher infection doses (Fig. 6B-D, Supplemental Fig. 4B,C), while Mip-1β was consistently higher in TIV/Cal08 mice regardless of infection doses (Supplemental Fig. 4D). As an alternative approach to increase NNAbs, TIV-mice were boost-immunized prior to Cal08-infection. Despite a significant increase in Bris59-specific HI titers and Cal08-binding NNAbs measured by ELISA (Supplemental Fig. 4E), A(H1N1)pdm09 morbidity based on BW changes was also not exacerbated (data not shown). Therefore, higher NNAb/virus ratios were not associated with morbidity of TIV/Cal08 mice during sub-lethal infection.

### NNAbs potentiated recruitment and activation of memory CD8 T cells in TIV/Cal08 mice

**Figure 7:**
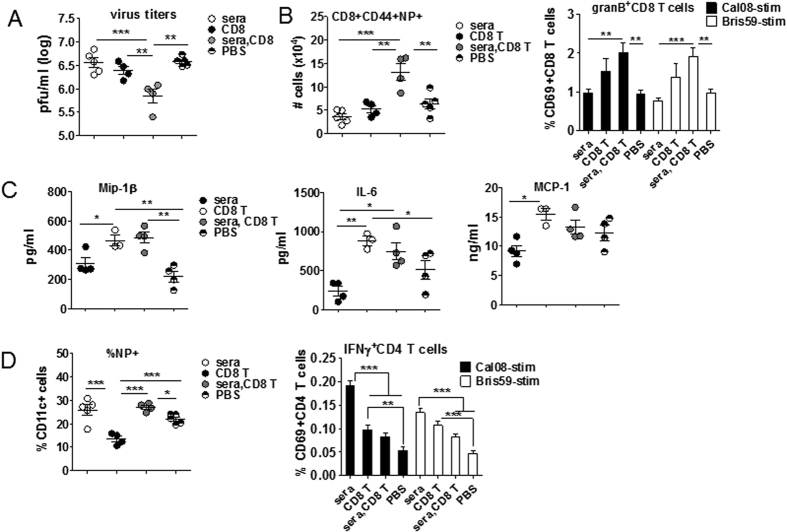
NNAbs potentiated recruitment and activation of memory CD8 T cells in TIV/Cal08 mice. Splenocytes and sera were collected from TIV (9 μg)-immunized Balb/c mice (10 mice/group) at d > 30 post-immunization and pooled. CD8 T cells were isolated via MACS and adoptively transferred to naïve Balb/c mice (1 × 10^7^ cells/mouse) with or without 200 μl sera (4–5 mice/recipient group). One day following adoptive transfer, recipients were infected with 5 × 10^6^pfu/mouse Cal08 virus. Control mice were PBS-transferred and then infected. All mice were sacrificed at d5 post-infection. (**A**) Lung virus titers were analyzed via plaque assay. (**B**) Lung cells were stained with NP pentamers to measure virus-specific CD8 T cells or *in vitro* stimulated with Cal08 or Bris59 virus at MOI 1 overnight to measure granB-secreting CD8 T cells. (**C**) Pro-inflammatory mediators (Mip-1β, IL-6, MCP-1) in lung lysates were measured via Bio-Plex^TM^. (**D**) Lung cells were intracellularly stained with NP to measure CD11c^hi^ cells containing NP signals. Lung cells were *in vitro* stimulated with Cal08 or Bris59 virus at MOI 1 overnight to measure IFNγ-secreting CD4 T cells via flow cytometry. The error bars represent standard error of the mean (SEM). One-way ANOVA with Bonferroni post-test was used to calculate the *P* values. *P < 0.05; **P < 0.01; ***P < 0.001. The data are a representative of 2 experiments.

In contrast to the initial hypothesis, our findings so far suggest that TIV-immune mice are protected through mechanisms involving NNAbs, virus-specific CD8 T cells and FcR-bearing phagocytic cells, similar to heterosubtypic immunity^34^,^39^,^40^,^41^,^42^. To delineate the key contributors for potent CD8 T cell response in TIV/Cal08, but not in PBS/Cal08 mice in the current context, sera or splenic CD8 T cells from TIV-mice were adoptively transferred into naïve mice (Fig. 7). For each naïve mouse that received 1 × 10^7^ isolated CD8 T cells, NP-specific and HA-specific CD8 T cells were 0.52% (5 × 10^4^) and 0.12% (1 × 10^4^) CD8 T cells, respectively, based on tetramer staining. All mice were then infected the next day with Cal08 virus. Efficient reduction of lung virus titers at 5 dpi required both sera and CD8 T cells, which was also correlated with highest activation of NP-specific CD8 T cells and their maximum granB-secretion (Fig. 7A,B, Supplemental Fig. 5A). These data indicate that both NNAbs and memory CD8 T cells are required for efficient protective immunity, with NNAbs potentiating CD8 T cell responses. Interestingly, CD8 T cells alone were sufficient to induce the majority of pro-inflammatory mediators as well as IL-10, while both sera and CD8 T cells were required for full IL-1β induction (Fig. 7C, Supplemental Fig. 5B). This indicates that CD8 T cell-mediated killing, rather than infection-induced cell death, is a trigger/amplifier of these proteins. The presence of sera was associated with intracellular NP signal within CD11c^hi^ cells (Fig. 7D) and CD11b^+^ monocytes (Supplemental Fig. 5C), indicating that accumulation of intracellular viral Ag is facilitated by NNAbs. While intracellular viral Ag could represent intracellular infection or receptor-mediated uptake, transfer of sera alone led to superior CD4 T cell recall responses (Fig. 7D) upon *in vitro* stimulation, supporting the latter scenario. It also confirms that NNAbs themselves do not exhibit pathological activity. Collectively, these data suggest that NNAbs cooperate with FcR-bearing cells to activate CD8 T cells.

## Discussion

An important observation from the Canadian sentinel reports is that despite the association of seasonal TIV with increased risk of A(H1N1)pdm09 infection, disease severity measured by hospitalization and death was not exacerbated^12^,^13^. Although our study is not set to test the increased risk of A(H1N1)pdm09 infection among TIV-immune mice, our findings show that when TIV-immune mice were infected with Cal08 virus, the disease measured by morbidity and mortality was not worsened. While animal studies utilizing pigs and ferrets^22^,^28^ have suggested that prior vaccination was associated with enhanced respiratory disease, our findings contrast with them in that TIV did not enhance disease and that viral titers were reduced by TIV-immunization even though TIV-sera exhibited no detectable neutralizing activity against Cal08 (Figs 1F, 4C, 5A, 6A and 7A). The reason for discrepancy is not entirely clear, but it is noteworthy that WIV was used as immunogen and lung virus replication was not directly measured in the pig model^22^ and that TIV did not elicit detectable HI titers, but only ELISA-measurable Abs in the ferret model^28^.

The balance between immune protection vs. immunopathology of virus-specific CD8 T cells during influenza virus infection determines the disease outcome^43^. There is ample evidence showing protection^44^,^45^,^46^,^47^,^48^ mediated by CD8 T cells as well as immunopathology, especially when regulatory mechanisms including costimulatory/inhibitory signals, are altered^49^,^50^,^51^,^52^,^53^,^54^,^55^. In our study, TIV/Cal08 mice showed overall better disease outcomes as measured by body weight, mortality, virus titers and viral clearance than PBS/Cal08 mice, instead of worsened disease outcomes. Importantly, these outcomes were associated with elevated inflammatory mediators and cytotoxic T cell activity in the lung. Thus, local lung injury and immunopathology may possibly have occurred and temporarily compromised the lung functions of TIV-immune mice. However, they did not lead to poor outcome, characteristic of immunopathology described in other animal studies following respiratory infections. Immune-regulatory mechanisms including IL-10R signaling may have prevented the weight loss. Multiple regulatory mechanisms appeared to operate in TIV/Cal08 mice, as αIL-10R-treated TIV/Cal08 mice, despite modest aggravation in morbidity during the 5 dpi, recovered at a faster rate than PBS/Cal08 mice during 14-days monitoring (data not shown). Thus, during the 2009 pandemic, immune-competent individuals such as middle-aged adults, even though mostly affected by A(H1N1)pdm09 infection, may have been able to prevent unchecked immunopathology, while individuals with immune-dysregulation may have been prone to severe outcomes. Interestingly, obesity (46%) was the most common underlying medical condition associated with A(H1N1)pdm09 fatal cases^56^, and recent studies linked obese conditions to a defect in IL-10 secretion by Treg cells via the insulin receptor-AKT/mTOR pathway^57^.

NNAbs are generally categorized as non-neutralizing by *in vitro* functional assays that do not encompass potential *in vivo* functions such as Ab-dependent cell-mediated cytotoxicity (ADCC) or complement-dependent cytotoxicity (CDC)^58^. These pathways, by engaging cellular components, can cause lysis of infected cells and induction of inflammation as mechanisms to limit virus replication. We have previously reported that non-hemagglutinating Abs can induce lysis of virus-infected MDCK cells by peritoneal inflammatory macrophages in an FcR-dependent manner^40^. In addition, recent studies by DiLillo and colleagues and by Tan and colleagues have demonstrated the significance of NNAbs against H1 and H7 for *in vivo* protection^59^,^60^. These NNAbs are raised against the HA head and are dependent on the Fc-FcγR interaction for *in vivo* protection, as in the case for broadly-neutralizing HA-stalk binding Abs^61^. Therefore, it is conceivable that NNAbs from TIV-immunized mice in our current study confer protection against Cal08 virus through ADCC by NK cells or macrophages. Notably, FcR levels were increased in the majority of CD11b^+^ cells in TIV/Cal08 mice (Supplemental Fig. 2A). However, no evidence of increased NK cell frequency or activation (IFNγ secretion) was observed by flow cytometry in the current study setting (data not shown). We also investigated the potential role of NNAbs for CDC in TIV/Cal08 mice. Although TIV/Cal08 mice secreted Abs (IgM and IgG3) that could potentially activate complement, complement-depletion did not directly affect BW, virus titers, NP-specific CD8 T cells or NP^+^ CD11c^hi^ cells (Supplemental Fig. 6). Nonetheless, it remains possible that these *in vivo* functions of TIV-sera may surface at a different stage of protective immunity or exhibit long-term impact. Indeed, recent studies have shown that ADCC-inducing Abs are readily detectable in normal human sera^62^, or increased following seasonal influenza vaccine as well as avian influenza vaccine^63^,^64^. Therefore, further investigation utilizing specific gene-knockout mice will help clarify crucial *in vivo* CDC or ADCC functions of TIV-sera.

Apart from HA-reactive NNAbs, NNAbs raised against NP have also been shown to confer protection^29^,^30^,^32^,^33^. Because *in vivo* protection by NP immunization correlates with CD8 T cells in these studies, CD8 T cells are thought to be the sole effector mechanism. However, recent studies have shown that such protection following NP immunization is also dependent on B cells and FcR^34^,^65^,^66^. Therefore, unlike HA-reactive NNAbs, NP-reactive NNAbs may confer protection mainly by enhancing cross-presentation of FcR-bearing cells. This idea has been recently demonstrated by León and colleagues that NP-immune complexes facilitate a prolonged antigen presentation by DCs and promote efficient memory CD8 T cell formation during the primary and secondary infection^35^. Our current findings are in line with these works that pre-existing NNAbs facilitate cross-presentation of internalized Ags to CD8 T cells, that are otherwise induced at significantly lower levels upon Cal08 infection in naïve mice (Fig. 7B, Supplemental Fig. 5A). Virus-specific CD8 T cells were in turn, directly associated with reduced virus titers (Fig. 7A). Under these conditions, the overall Ag-presentation machinery of CD11c^hi^DCs was significantly enhanced as shown by upregulation of FcR, MHC class I (H-2^d^) and MHC class II (I-A^d^) expression (Fig. 5C, Supplemental Figs 2A and 3B) in addition to the induction of pro-inflammatory cytokines (Fig. 2A,D). NP-reactive NNAbs may be poorly induced by TIV, and are rarely boosted by seasonal influenza vaccination in human^31^. In our current study, not all NNAbs of TIV-immunized mice were directed against HA (Fig. 1B,C), yet these NNAbs may be sufficient enough to induce potent CD8 T cell responses. Collectively, these data suggest a clinical potential of NP-reactive NNAbs for cross-protection against not only seasonal influenza but also A(H1N1)pdm virus infection.

Apart from FcR dependency for *in vivo* protection, neutralizing vs. NNAbs may lead to qualitatively different outcomes in the cross-presentation process, as shown by the sharp difference in virus-specific CD8 T cell responses as well as CD11c^+^ cells between TIV/Cal08 vs. TIV/Bris59 mice (Figs 2C and 3C,D). Regardless of neutralizing activities, ICs would be internalized by phagocytic cells through Fc-FcR interaction. Intracellular signals regulating the cross-presentation process remains largely unknown, but studies have suggested that internalization alone is not sufficient for cross-presentation and may require additional intracellular pathways for efficient cross-presentation^67^. In addition, Ag-binding induces a conformational change in the heavy chain constant region of Ab^68^. Therefore, NNAbs-FcR interaction may transduce differential intracellular signals from neutralizing Abs-FcR interaction, which allow efficient cross-presentation. Interestingly, recent studies have shown that DCs can provide qualitatively different activation signals to virus-specific CD8 T cells in an epitope-specific manner^69^. Considering the role of the NP-immune complex for efficient cross-presentation^35^, it is tempting to speculate a correlation between NNAbs raised at different levels during the primary response and levels of CD8 T cell activation. Among FcR-bearing cells, both CD11c^hi^ DCs and alveolar macrophages appear to promote cross-presentation to CD8 T cells. DCs are the most efficient cells for cross-presentation *in vivo*, thanks to reduced proteolysis compared to macrophages^67^,^70^. However, other studies have identified the role of macrophages for CD8 T cell activation^42^, while our current findings (Figs 3B, 5C and 7D) and recent studies^71^ support the role of CD11c^hi^ DCs.

A potential role of NNAb in TIV/Cal08 mice for ADE was also suspected, as intracellular NP signals in CD11c^hi^ DCs (Figs 3B and 7D) and simultaneous activation of virus-specific CD8 T cells (Fig. 3C,D), could represent an enhanced intracellular infection following NNAbs-mediated internalization, as described for dengue or HIV infections^72^. However, recent studies have shown that it is the endocytosed exogenous virus particles rather than intracellular infection that leads to significant cross-presentation in myeloid DCs^73^. In addition, opsono-phagocytosis of ICs by macrophages significantly contributed to the viral clearance of influenza^74^. To address whether intracellular infection occurs following FcR-mediated Ag-uptake in DCs or macrophages, mixtures of serially diluted TIV-sera and Cal08 or Bris59 virus (2 × 10^3^TCID50/ml) were incubated with bone marrow-derived DCs or macrophages (BM-Mɸ) overnight and intracellular virus replication was assessed. While both viruses were able to infect BM-Mɸ (Supplemental Fig. 7A,B) or DCs (data not shown), the presence of TIV-sera did not increase M-gene expression compared to the virus control, or sustained intracellular infection during longer incubation. Suppression of virus titers were associated with induction of anti-viral responses including tripartite motif-containing protein 21 (TRIM21)^75^, retinoic acid-induced gene-I (RIG-I)^76^ genes and IFNβ secretion in a sera Ab dose-dependent manner (Supplemental Fig. 7C). Thus, FcR-bearing cells of TIV/Cal08 mice may be resistant to intracellular infection upon internalization of ICs, but are poised to readily promote an anti-viral state. In agreement with this idea, recent studies have shown that influenza replication is abortive in DCs^77^. These features of FcR-bearing cells may have prevented ADE in TIV/Cal08 mice.

Our current findings provide significant insights into the interaction between pre-existing immunity and pandemic influenza virus. By definition, pandemic virus exploits the lack of neutralizing Abs in the general population, yet has to face pre-existing NNAbs and memory CD8 T cells that can play a significant role in determining disease outcome, apart from virulence of the pandemic virus itself. The current study highlighted the role of seasonal vaccine-induced NNAbs for protection against A(H1N1)pdm09 infection rather than enhancement of disease, through the mechanisms engaging FcR-bearing cells and pre-existing CD8 T cells.

## Methods

### Mice, cells and viruses

Balb/c mice were purchased from Jackson Laboratory. Mouse handling, bleeding, infection and immunization were previously described^78^. Collection of tissues including spleen and lungs was performed after euthanizing mice with a lethal dose of Avertin (Sigma-Aldrich). Bronchoalveolar lavage (BAL) was collected by injecting 1 ml PBS +0.5% bovine serum albumin (BSA) through the trachea with an 18G catheter. Nasal washes were collected by passing 0.5 ml PBS +0.5% BSA through the nasal passage. Madin-Darby canine kidney (MDCK) cells were maintained in Dulbecco’s modified Eagle’s medium containing antibiotics, glutamine and 10% fetal bovine serum and used for plaque assays as previously described^78^. Influenza viruses were propagated in 11 day-old embryonic chicken eggs and clarified allantoic fluid was collected as previously described^78^. This study was approved by CDC Institutional Animal Care and Use Committee and was conducted in an Association for Assessment and Accreditation of Laboratory Animal Care International accredited animal facility. This study was carried out in strict accordance with the Animal Welfare Act regulations of the United States Department of Agriculture and Public Health Service Policy on Humane Care and Use of Laboratory Animals, administered by the National Institutes of Health. All efforts were made for animal welfare, including influenza virus infection under light anesthesia with Avertin and daily monitoring of body weight.

### Adoptive transfer

TIV-immunized mice (at >d30 post immunization) were bled for collection of sera and then euthanized for collection of spleens. After single cell suspension, resting CD8 T cells were isolated by negative selection by magnetic-activated cell sorting (MACS; Miltenyi Biotec) and approximately 1 × 10^7^ CD8 T cells/200 μl PBS were injected through the tail-vein into naïve Balb/c mice. Pooled sera (200 μl/mouse) were injected i.v. into naïve mice. Sera-transferred mice were subsequently bled to evaluate the HI titers. One day following adoptive transfer, mice were infected i.n. with 5 × 10^6^pfu/mouse Cal08 virus. Five days following infection, lungs were collected for assessment of immune responses.

### Influenza vaccine, IL-10R blocking or isotype control Abs

TIV (2009–10; Sanofi-Pasteur) was injected i.m. into mice (100 μl/mouse). IL-10R blocking and rat IgG1 isotype control Abs (Biogend) were injected i.p. (0.5 mg/mouse) on days −1, +1 and +3 post-infection with influenza viruses.

### Assessment of Ab response and Ab-secreting cells (ASCs)

Microneutralization (MN), HI assay were previously described^78^. Sera and mucosal (BAL and nasal washes) Abs binding to influenza viruses were analyzed by ELISA. Briefly, Nunc 96 well plates (Maxi-sorb) were coated with 100 HAU WIV or 2 μg/ml His-tagged rHA proteins, then blocked with 4% BSA in PBS-Tween for 1 hr. Two or ten-fold dilutions of samples are added to the plates for 2 hr at room temperature or overnight at 4 °C. Plates were then developed by biotin-anti-mouse IgG/IgA followed by streptavidin (SA)-HRP (Southern Biotech). The signals were developed using 1 x TMB (ebioscience) and measured at 450 nm using a plate reader (Biotek). Ab binding strength was measured by biolayer interferometry on an Octet Red instrument (Fortebio, Inc.) using H1N1 recombinant HAs as previously described^79^. The frequency of ASCs in spleen was measured by ELISpot assay as previously described^80^. Briefly, ELISpot plates (Millipore) were coated with 100 HAU WIV overnight and blocked with cRPMI-1640 media. Dilutions of cells were added to plates and incubated overnight at 37 °C. Plate-bound Abs were probed by biotin-anti-mouse IgG, SA-alkaline phosphatase (Southern Biotech), then Vector Blue Substrate Kit (Vector Lab). Spots were counted using an ImmunoSpot^®^ ELISPOT reader (Cellular Technology Ltd.).

### Assessment of inflammatory mediators in lung lysates and lung histology sections

Pro-inflammatory mediators in lung lysates were analyzed using Bio-Plex Pro^TM^ mouse Cytokine Assay-7-plex on a Bio-Plex System (Bio-Rad) according to the manufacturer’s instructions. IP-10 was measured using IP-10 ELISA kit (Abcam). For histology and immunochemistry of lung sections, mouse lungs were fixed by perfusion with 10% formalin (W/V) via the vena cava. After removal, lungs were placed in 70% ethanol before routine paraffiin embedding and processing into 0.5 mm sections. Lung histology and immunochemistry were performed on 10% formalin (W/V)-fixed sections by staining with hematoxylin and eosin (H&E) or mouse anti-NP (CDC scientific resources), biotinylated α−mouse IgG and hematoxylin (Biocare). Whole slides were scored based on lymphocyte infiltration as well as small airway and bronchiolar inflammation.

### Monoclonal Ab staining and Flow Cytometry

Cells were stained with CD11b, CD95, CXCR5, CD3, CD8a, CD44 (BD Bioscience); CD103, I-A^d^, GL7, CD138, CD69, CD86, CD19, CD16/32, Ly6C (Biolegend); CD49b, F4/80, CD11b, CD11c, CD45, PD-1, H-2^d^ (eBioscience). Virus-specific CD8 T cells were identified using H-2K^d^/IYSTVASSL (HA) and H-2K^d^/TYQRTRALV (NP) pentamers (Proimmune). For *ex vivo* staining of cytokine-secreting cells, cells were stained first with surface markers then fixed/permeabilized for intracellular cytokine staining. For *in vitro* stimulation, cells were infected with Bris59 or Cal08 virus at a multiplicity of infection (MOI) of 1 for 1 hr. After incubation overnight and addition of Golgi-Plug (BD) for the last 6 hr, cells were stained for CD4 and CD8, permeabilized and stained for IFNγ, IL-2, TNFα, granzyme B, IL-10, IL-4 or IL-6. Cells were analyzed with a FORTESSA flow cytometer (BD) and FlowJo software (Tree Star, Inc.).

### Assessment of antibody-dependent enhancement of infection using bone marrow-derived macrophages (BM-Mɸ) culture

Mouse BM cells were incubated with recombinant M-CSF (2 ng/ml) for 7 days with media replenishment on d4. For *in vitro* assessment of ADE, serial dilutions (1:40 through 1: 1280) of sera from TIV-immunized mice were incubated with 2 × 10^3^TCID/ml Bris59 or Cal08 virus for 2 hr, then placed onto BM-Mɸ overnight. Culture supernatants were analyzed for type I IFN by ELISA and cells were lysed for qPCR for the M gene. Alternatively, TIV-sera and virus mixtures were added to cells for 1 hr, then unbound or free viruses were washed away prior to incubation for 3 days. The viruses in the supernatants were assessed at d3 via plaque assay.

### Statistics

One-way ANOVA analysis with a Bonferroni post-test was used for comparison of multiple groups. For comparison between Cal08- vs. Bris59-responses within the group, a student t-test was used. For analysis of body weight (BW) changes, two-way ANOVA with Bonferroni’s multiple comparison test was used. For statistical designations, *denotes *P* < 0.05; **denotes *P* < 0.01; ***denotes *P* < 0.001.

## Additional Information

**How to cite this article**: Kim, J. H. *et al.* Non-neutralizing antibodies induced by seasonal influenza vaccine prevent, not exacerbate A(H1N1)pdm09 disease. *Sci. Rep.* **6**, 37341; doi: 10.1038/srep37341 (2016).

**Publisher’s note**: Springer Nature remains neutral with regard to jurisdictional claims in published maps and institutional affiliations.

## Supplementary Material

Supplementary Information
