# Supplementary material for: Non-neutralizing antibodies induced by seasonal influenza vaccine prevent, not exacerbate A(H1N1)pdm09 disease
Source: Sci Rep. 2016 Nov 16;6:37341. doi: 10.1038/srep37341 (PMC5110975; doi:10.1038/srep37341)
Supplement: Supplementary Information [file srep37341-s1.pdf]

Non-neutralizing antibodies induced by seasonal influenza vaccine prevent, not exacerbate A(H1N1)pdm09 disease

Jin Hyang Kim[1*], Adrian J. Reber[1], Amrita Kumar[1], Patricia Ramos[1], Gabriel Sica[2], Nedzad Music[1], Zhu Guo[1], Margarita Mishina[1,3], James Stevens[1], Ian A. York[1], Joshy Jacob[4], Suryaprakash Sambhara[1]

Supplemental Information

Supplemental Figures and Figure legends

Supplemental Fig. 1

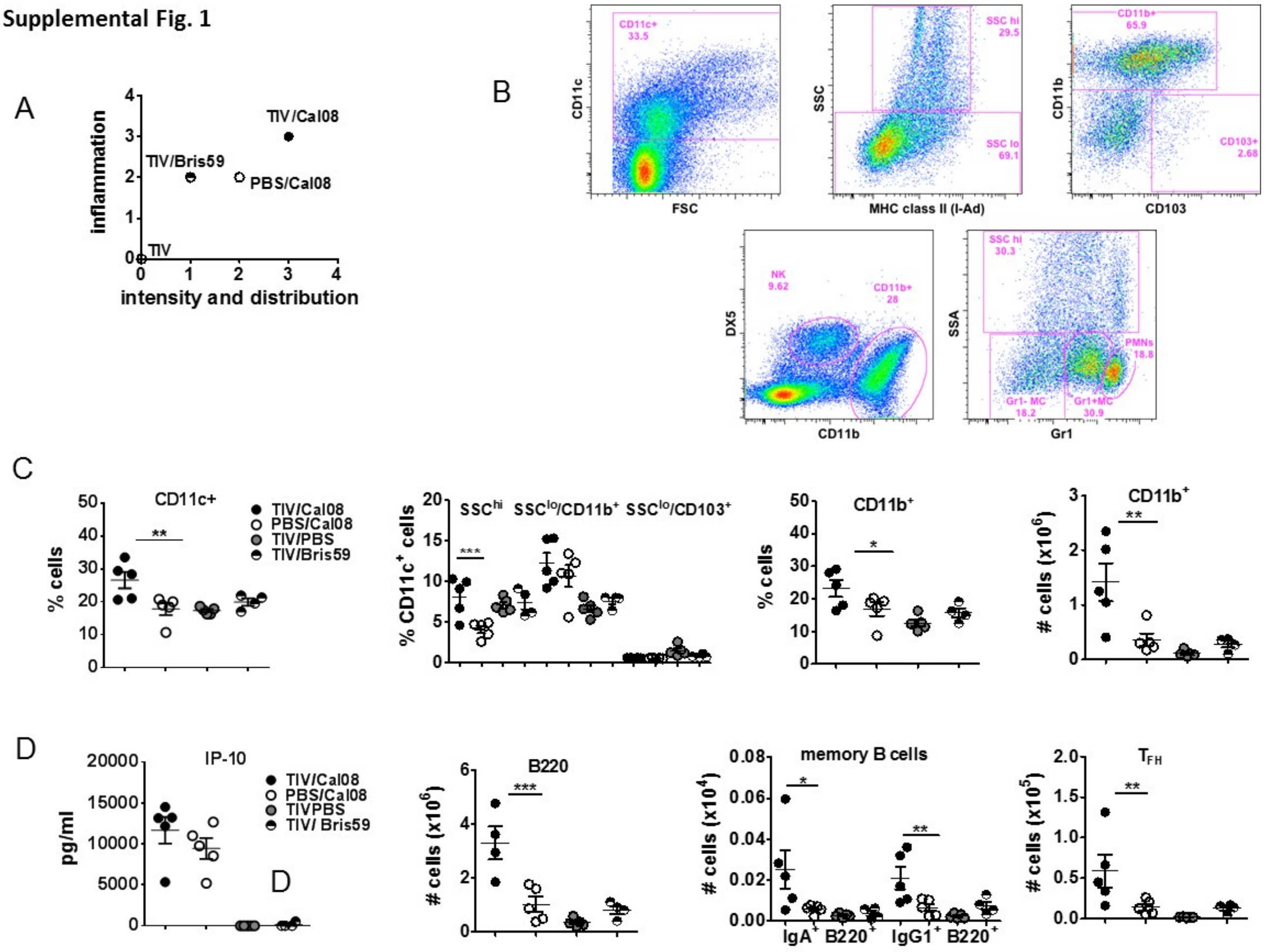

Supplemental Figure 1. **Cell infiltration was significantly elevated in TIV/Cal08 mice.** (A) H&E stained lung sections were scored for overall lung inflammation on a scale of 1 through 4, based on small airway inflammation (mononuclear cells, eosinophils, plasmacytoid cells, intraepithelial lymphocytes in bronchiolar submucosa and epithelial damage) and intensity and perivascular distribution of inflammatory cells and parenchymal cell injury. (B) Lung single cell suspensions were stained for flow cytometric analysis at d5 post-infection. Gating strategy for $CD11c^+$ and their subsets (SSC^{hi}, $SSC^{lo}/CD11b^+$, $SSC^{lo}/CD103^+$), NK cells ($DX5^+$), $Gr1^+$inflammatory monocytes ($Gr1^+CD11b^+SSC^{int}$) and neutrophils ($CD11c^-CD11b^+Gr1^{hi}$) is shown. (C) The frequencies of $CD11c^+$ cells and their subsets, the frequency of $CD11b^+$ cells and their cell numbers are shown. (D) IP-10 was measured by ELISA, and B220 B cells,

IgA+ or IgG1+ memory B cells and CD4+CXCR5+PD1+ T_{FH} cells were measured by flow cytometry and their numbers were calculated from lung cell counts.

Supplemental Fig. 2

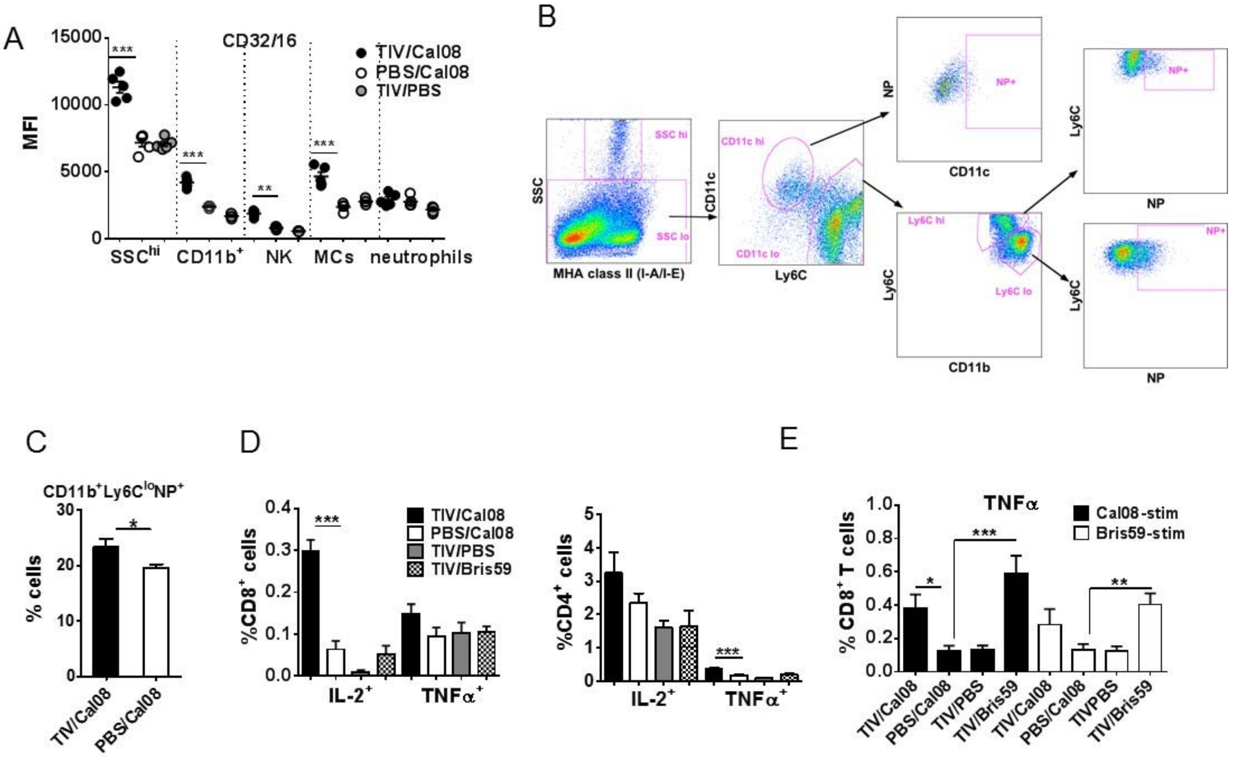

Supplemental Figure 2. The higher level of intracellular viral protein in phagocytic cells correlated with enhanced cellular immunity in TIV/Cal08 mice. (A) Lung single cell suspensions at 5dpi were stained for phagocytic cells including SSC^{hi}, SSC^{lo}/CD11b^{+}, NK cells (DX5^{+}), inflammatory monocytes (MCs) (Gr1^{+}CD11b^{+}SSC^{int}) and neutrophils (CD11c^{-}CD11b^{+}Gr1^{hi}) for their expression of CD32/16. (B) The gating strategy to identify intracellular NP positivie-CD11c^{hi}, Ly6C^{hi}CD11b^{+}, Ly6C^{lo}CD11b^{+} cells is shown. (C) The frequency of Ly6C^{lo}CD11b^{+} cells that were stained with NP was compared between TIV/Cal08 and PBS/Cal08 mice. (D) Frequencies of lung CD8 or CD4 T cells secreting IL-2 or TNFα *ex vivo* were analyzed by flow cytometry. (E) Frequency of TNFα-secreting CD8 T cells in response to Cal08 or Bris59 *in vitro* at MOI 1 overnight was analyzed by flow cytometry.

Supplemental Fig. 3

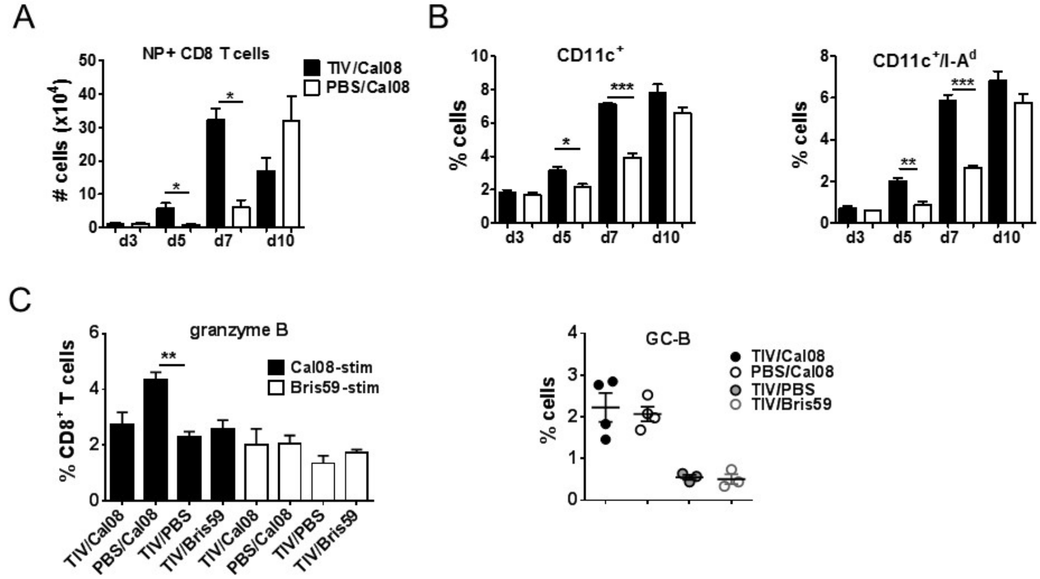

Supplemental Figure 3. Homeostasis of adaptive immunity of TIV/Cal08 mice was established following accelerated kinetics of cellular immune activation. TIV- or mock-immunized Balb/c mice were infected with $5x10^6$pfu/mouse Cal08 virus. (A) Lung cells were stained and analyzed for the frequency or numbers of NP-specific CD8 T cells at 5dpi. (B) $CD11c^{hi}$ cells contained intracellular NP or upregulating MHC class II ($I\text{-}A^d$) were analyzed via flow cytometry. (C) Splenocytes from TIV/Cal08, PBS/Cal08, TIV or TIV/Bris59 mice at 21dpi were *in vitro* stimulated with Cal08 or Bris59 virus at MOI 1 overnight to measure granB-secreting CD8 T cells via flow cytometry or *ex vivo* stained for the frequency of GC-B cells ($GL7^{hi}CD95^{hi}$).

Supplemental Fig. 4

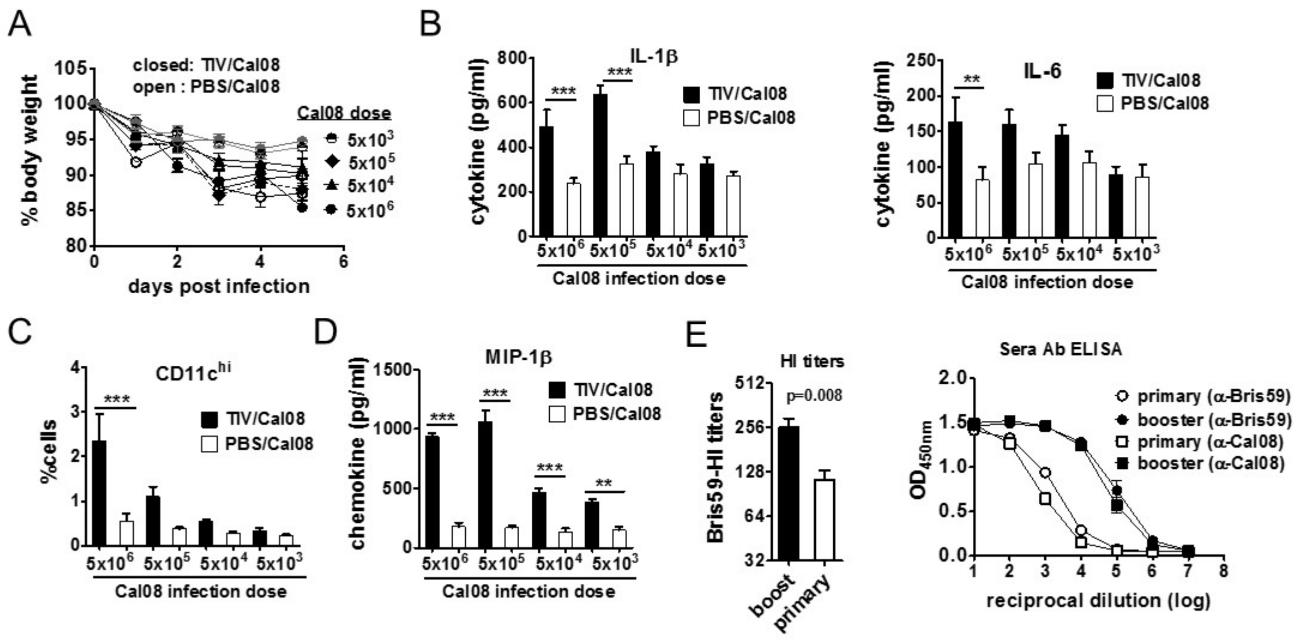

Supplemental Figure 4. NNAbs did not correlate with potential vaccine-associated enhanced disease. TIV or mock-immunized Balb/c mice were infected with 5x10^3-5x10^{6}pfu/mouse Cal08 virus in 1 log increments at d>30 post-immunization. (A) BWs were monitored during 5 days post-infection. (B) Lung lysates at 5dpi were analyzed for the level of pro-inflammatory mediators, IL-1β and IL-6 via Bio-Plex^{TM}. (C) Lung cells were intracellularly stained with NP and %CD11c$^+$ cells with intracellular NP signals are shown. (D) Lung lysates at 5dpi were analyzed for the level of Mip-1β via Bio-Plex^{TM}. (E) TIV-immunized mice were booster-immunized with 3μg TIV and sera collected at >30 days post-booster immunization were tested for Bris59-specific HI titers and Cal08-binding Abs by ELISA.

Supplemental Fig. 5

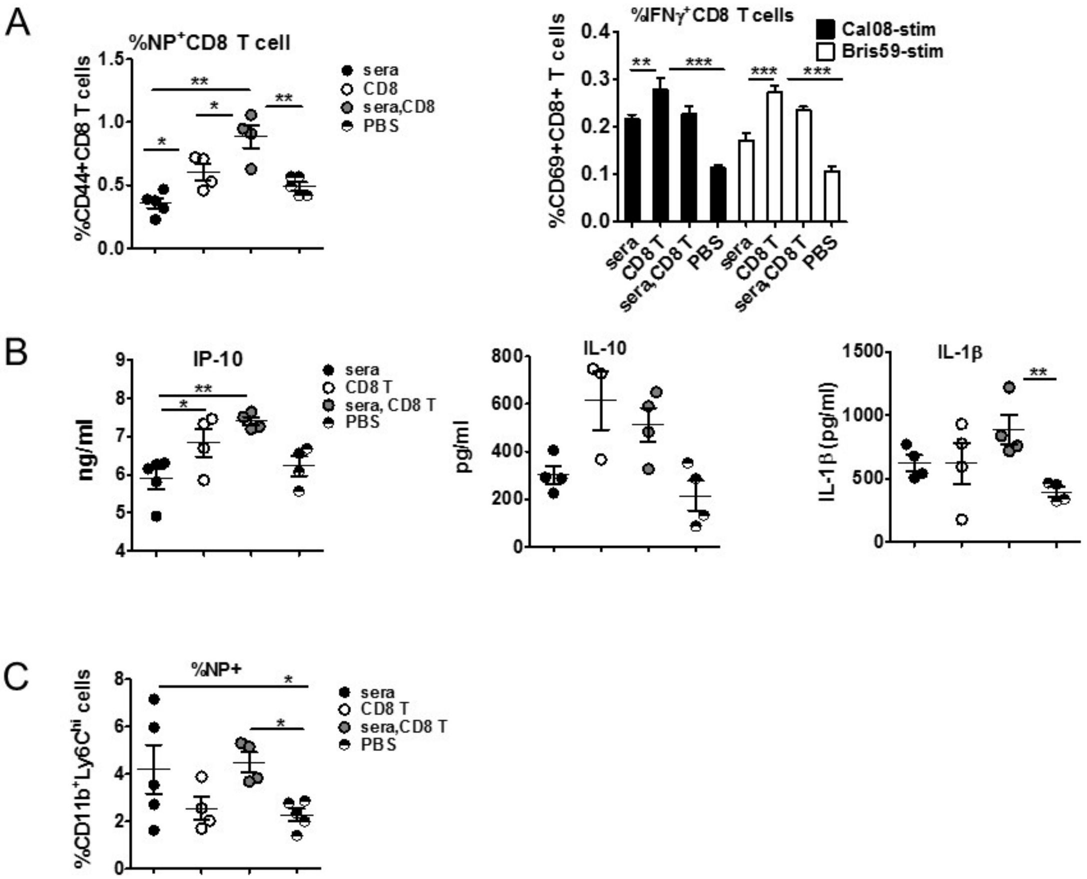

Supplemental Figure 5. **NNAbs potentiated recruitment and activation of CD8 T cells upon Cal08 infection**. Splenocytes and sera were collected from TIV (9µg)-immunized Balb/c mice at d>30 post-immunization and pooled. CD8 T cells were isolated via MACS and adoptively transferred to naïve Balb/c mice ($1x10^7$ cells/mouse) with or without 200µl sera. One day following adoptive transfer, recipients were infected with $5x10^6$pfu/mouse Cal08 virus. Control mice were PBS-transferred and then infected. (A) At 5dpi, lung single cell suspensions were stained with NP pentamers to measure virus-specific CD8 T cells or *in vitro* stimulated with Cal08 or Bris59 virus at MOI 1 overnight to measure IFNγ-secreting CD8 T cells via flow cytometry. (B) Pro-inflammatory mediators (IP-10, IL-1β) or the anti-inflammatory mediator (IL-10) in lung lysates were measured via ELISA or Bio-Plex™. (C) Lung cells were intracellularly stained for NP to measure $CD11b^+Ly6C^{hi}$ cells containing NP signals.

Supplemental Fig. 6

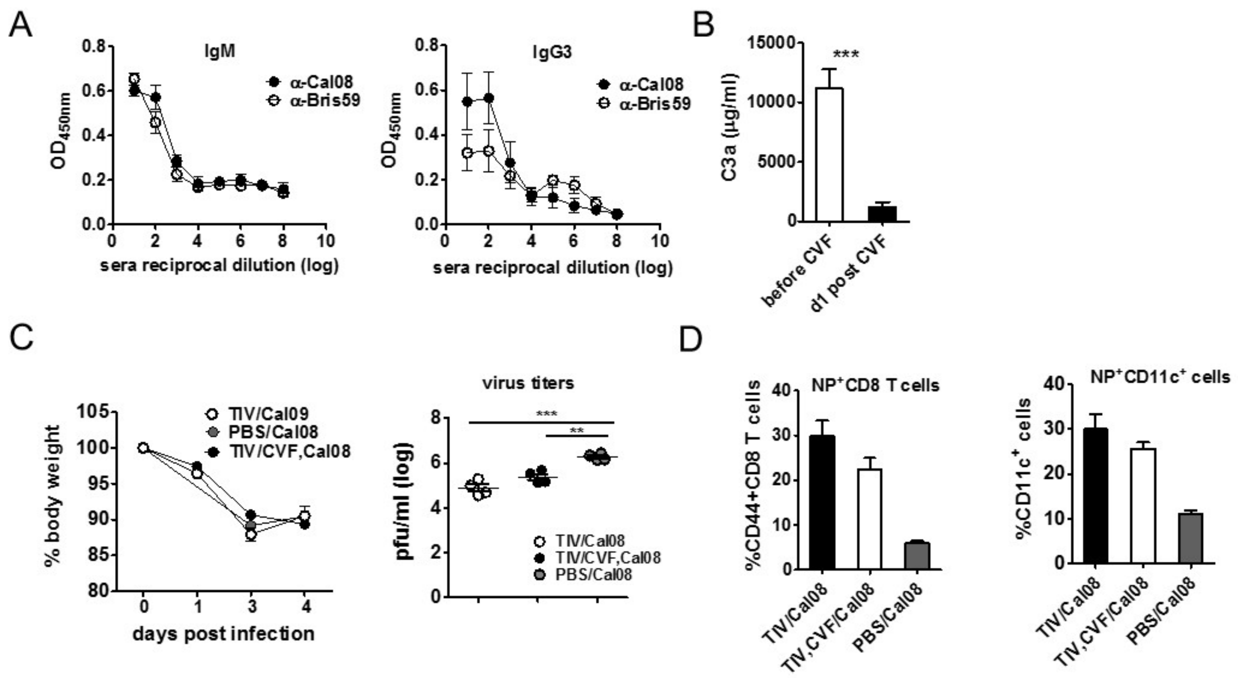

Supplemental Figure 6. Complement depletion did not affect morbidity or virus titers in TIV/Cal08 mice. (A) Sera collected from TIV-immunized mice at d>30 post-immunization were analyzed for the amount of IgM and IgG3 specific to Cal08 or Bris59 virus via ELISA. (B) TIV-immunized mice were injected with 50µg Cobra Venom factor (CVF) at d1 and 4hr prior to Cal08-infection and complement depletion was verified by measuring sera C3 levels at d1 post-CVF treatment via ELISA. (C) All mice were infected with 5x10^6pfu Cal08 virus and BWs were monitored for 5 days post-infection and lung virus titers at 5dpi were measured via plaque assay. (D) Lung cell suspensions were stained with NP pentamers to measure NP-specific CD8 T cells or CD11c^{hi} cells stained with intracellular NP.

Supplemental Fig. 7

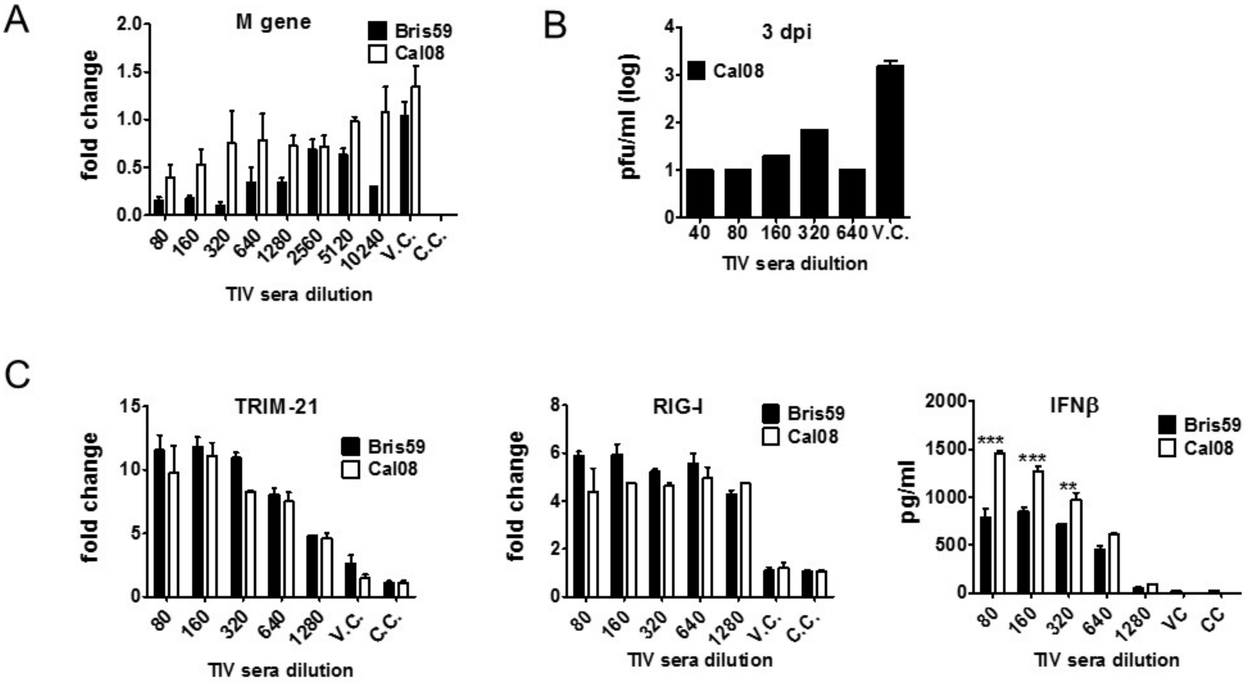

Supplemental Figure 7. TIV-sera did not induce ADE in bone marrow-derived macrophages. Mouse BM cells were incubated with rM-CSF (2ng/ml) for 7 days with media replenishment on d4. For *in vitro* assessment of ADE, serial dilutions (1:40 through 1: 1280) of pooled TIV-sera were incubated with $2x10^3$TCID/ml Bris59 or Cal08 virus for 2 hr, then placed onto BM-Mϕ overnight. (A) Cells were then washed and lysed for mRNA extraction and viral replication was measured by qPCR for M gene expression. (B) For assessment of intracellular replication of virus, BM-Mϕ cells were incubated with pooled TIV-sera + virus mixture for 1hr for viral attachment and then free viruses in the supernatant were removed. Cells were then incubated for 3 days and the supernatants were collected for virus titers via plaque assay. (C) To assess the anti-viral state of BM-Mϕ following infection, mRNA extracts from (A) were further measured for TRIM-21 and RIG-I. Culture supernatants were collected for measurement of IFNβ via ELISA. Virus control (V.C.) refers to cells infected without sera and cell control (C.C.) refers to cells non-infected.
